# Isolation and characterization of *Sphingomonadaceae* from fouled membranes

**DOI:** 10.1038/s41522-018-0074-1

**Published:** 2019-01-25

**Authors:** Hendrik J. de Vries, Florian Beyer, Monika Jarzembowska, Joanna Lipińska, Paula van den Brink, Arie Zwijnenburg, Peer H. A. Timmers, Alfons J. M. Stams, Caroline M. Plugge

**Affiliations:** 1 Laboratory of Microbiology, Wageningen University & Research, Stippeneng 4, 6708 WE Wageningen, The Netherlands; 2 Wetsus, European Centre of Excellence for Sustainable Water Technology, Oostergoweg 9, 8911 MA Leeuwarden, The Netherlands

## Abstract

Membrane filtration systems are widely applied for the production of clean drinking water. However, the accumulation of particles on synthetic membranes leads to fouling. Biological fouling (i.e., biofouling) of reverse osmosis and nanofiltration membranes is difficult to control by existing cleaning procedures. Improved strategies are therefore needed. The bacterial diversity on fouled membranes has been studied, especially to identify bacteria with specialized functions and to develop targeted approaches against these microbes. Previous studies have shown that *Sphingomonadaceae* are initial membrane colonizers that remain dominant while the biofilm develops. Here, we characterized 21 *Sphingomonadaceae* isolates, obtained from six different fouled membranes, to determine which physiological traits could contribute to colonization of membrane surfaces. Their growth conditions ranged from temperatures between 8 and 42 ^o^C, salinity between 0.0 and 5.0% w/v NaCl, pH from 4 and 10, and all isolates were able to metabolize a wide range of substrates. The results presented here show that *Sphingomonadaceae* membrane isolates share many features that are uncommon for other members of the *Sphingomonadaceae* family: all membrane isolates are motile and their tolerance for different temperatures, salt concentrations, and pH is high. Although relative abundance is an indicator of fitness for a whole group, for the *Sphingomonadaceae* it does not reveal the specific physiological traits that are required for membrane colonization. This study, therefore, adds to more fundamental insights in membrane biofouling.

## Biological fouling: Physiology of Sphingomonadaceae in fouled filtering membranes

Water filtration systems inevitably become colonized by a diverse range of microorganism; an early colonizer, *Sphingomonadaceae*, is now shown to be uncommonly motile and have high tolerance to changes in temperature, salt concentration and pH when growing in biofouled membranes. Although bacterial diversity in biofouled membranes has been studied extensively, targeted strategies to prevent or reverse colonization are still lacking. Hendrik J. de Vries and colleagues studied 21 *Sphingomonadaceae* isolates from six fouled membranes, and observed a higher tolerance to growth in temperatures between 8 and 42 °C, 0.0 to 5.0% w/v NaCl, and pH between 4 and 10, as well as an increased twitching motility and active pili. These adaptive physiological properties are thought to be advantageous by facilitating membrane surface colonization, and may explain why *Sphingomonadaceae* are resistant to fouled membrane cleaning procedures.

## Introduction

The demand for high-quality water has increased in recent years and will rise even more in the future.^1,2^ Membrane filtration systems are attractive technologies to purify water: their high efficiency to separate water from its solutes delivers the option to remove most contaminants including pharmaceutical remnants within a single purification step in a relatively cost effective manner.^1^ Different membrane types have different separation properties and membranes can, therefore, be used in many applications.^3^ Low-pressure membranes (i.e., microfiltration and ultrafiltration) separate via pore-separation, while in high-pressure membranes (i.e., nanofiltration (NF) and reverse osmosis (RO)) separation occurs via dissolvent and diffusion processes (e.g., solution-diffusion model).^4^ Membrane filtration has one major disadvantage: fouling.^5^ Pre-treatment of the influent and periodical chemical cleaning of the membrane are, therefore, needed to control membrane fouling.^6,7^ Pre-treatment for high-pressure membranes is conventionally performed using a combination of processes, such as coagulation and flocculation followed by granular media filtration (e.g., anthracite coal, silica sand, or garnet) and cartridge filtration.^8^ Low-pressure membranes (microfiltration and ultrafiltration) filtration systems provide better removal efficiency compared to conventional pre-treatment systems but high capital and operation costs have hampered their implementation in the past.^8^ Chemical cleaning leads to a reduction in membrane lifetime and does not restore membrane performance completely under most circumstances (depending on the fouling type).^5^ The lack of alternatives makes chemical cleaning inevitable yet more effective and economically feasible antifouling strategies are needed.

Biofilm formation on the membrane surface leads to biological fouling (biofouling).^9^ Compared to other fouling types (colloidal matter, scaling, and organic fouling) biofouling is difficult to prevent or control because micro-organisms multiply and secrete extra-cellular polymeric substances (EPS) that protect a part of the microbial community against the chemical cleaning agents.^7,9^ Natural biofilms commonly consist of many different microbial species.^10^ In membrane biofilms, the microbial community composition is complex as well and is influenced by a variety of different parameters. Including the influent quality, pre-treatment steps of the feed water, local conditions such as temperature and seasonal change, the oxygen concentration in the influent, the organization of cascading membrane elements into vessels and stages and membrane cleaning.^6,11–15^ However, the significant change in microbial community diversity between free-floating bacteria present in the feed stream and membrane biofilms indicates that membrane filtration provides a selective force.^16^ Bacteria belonging to the phylum of *Proteobacteria*, particular those belonging to α-, ß-, and γ-lineage, have been shown to frequently dominate membrane biofilms.^6,11,14–18^

Yabuuchi et al.^19^ discovered an α proteobacterium that contained glycosphingolipids (GSL) in its cell envelope and proposed the genus *Sphingomonas* to accommodate this species. Takeuchi et al.^20^ classified the *Sphingomonas* species in four genera: *Sphingomonas sensu stricto, Sphingobium, Novosphingobium*, and *Sphingopyxis*. These genera, together with other newly discovered genera, now constitute the *Sphingomonadaceae*, a family that belongs to the class of α-*Proteobacteria*.^21^ It was found that *Sphingomonadaceae* initiate the formation of membrane biofilms and remain dominant during the biofilm maturation steps, both in spiral wound membranes and in membrane bioreactors, regardless of the surface properties of the membranes.^17,22,23^ Here, we describe the properties of 21 *Sphingomonadaceae* isolates, isolated from membrane surfaces used in full-scale operation and laboratory simulation experiments. We aimed to get insight into the physiological traits that determine their effective colonization of membrane surfaces.

## Results

### Isolation and identification

**Fig. 1:**
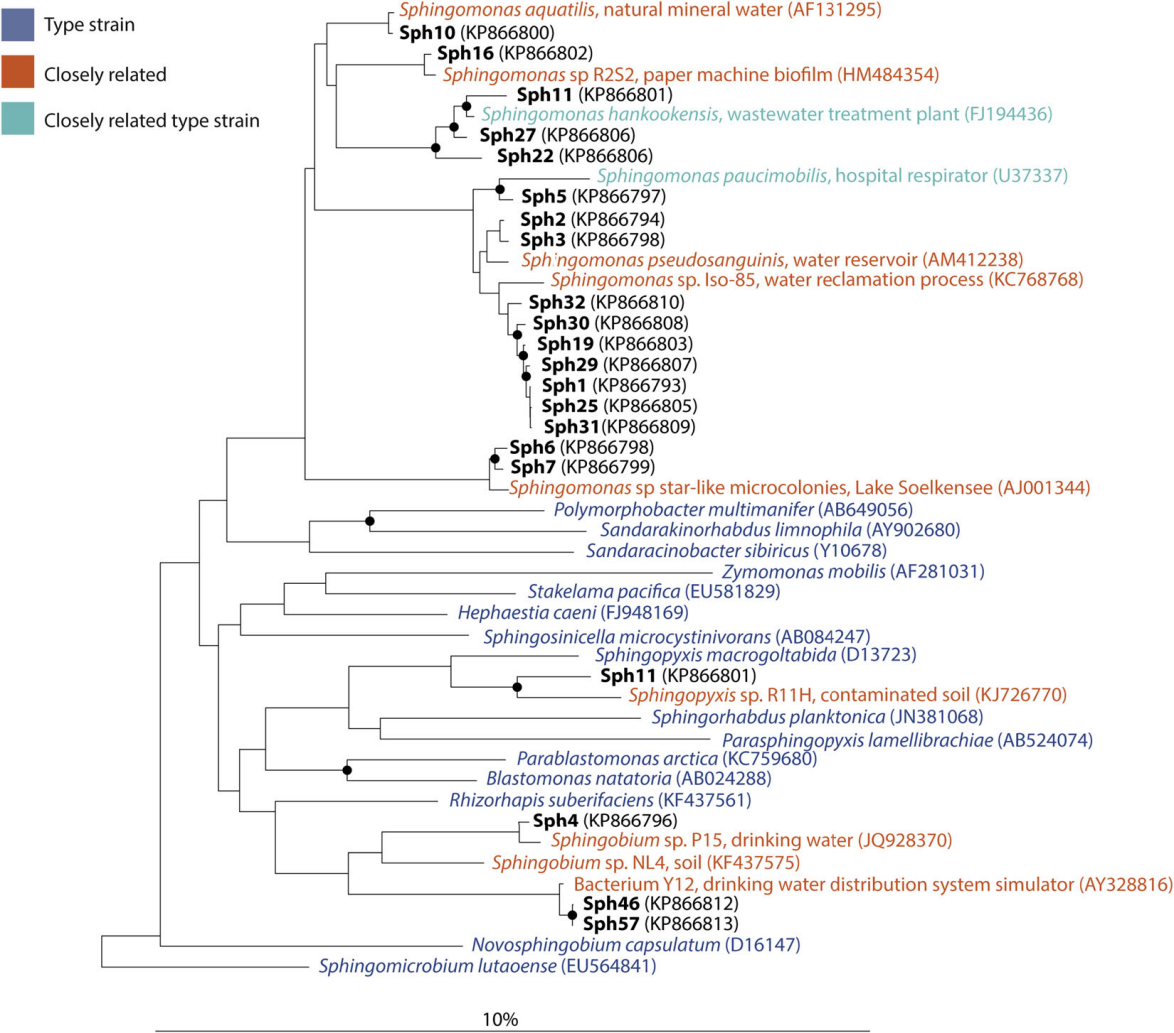
Phylogenetic tree inferred by the neighbor-joining method using almost complete 16S rRNA gene sequences derived from the SILVA SSU Ref database and from the Sph isolates (this study)

To study the behavior of bacteria relevant in membrane biofouling, we isolated 60 bacterial strains from 12 different membranes: ten used in full-scale operation and two in laboratory fouling simulation experiments. Because of the relevance of *Sphingomonadaceae* in membrane biofouling, the biomass obtained from the membranes was cultivated under conditions that select for *Sphingomonas*.^24^ Nearly full-length 16S rRNA gene sequences were obtained from the 60 isolates (sequences varying in length from 1245 – 1431 bp, except for Sph43 and Sph56, which had a sequence length of 791 and 796 bp, respectively). The strains were assigned using BLAST searches against the nucleotide database to find the closest relative of named sequences in the GenBank. Thirty-nine of the 60 isolates did not belong to the *Sphingomonadaceae* family but belonged to groups that are commonly found on fouled membranes, including *Actinobacteria* and *Bacteroidetes* (Supplementary Table 1).^25^ The 21 *Sphingomonadaceae* (Sph) isolates that were selected for further characterization were isolated from six different membranes (Table 1 and Supplementary note 1). In order to reveal the phylogenetic relationship between the Sph isolates, a phylogenetic tree was constructed based on the 16S rRNA gene sequences. This showed that the 21 Sph isolates clustered into 12 clades (a group of organisms considered as having evolved from a common ancestor) in the genera *Sphingomonas*, *Sphingopyxis*, and *Sphingobium* (Fig. 1). The members of each clade were isolated from the same membrane and, therefore, appeared to be paraphyletic (i.e., having a common evolutionary origin; Table 1). As identical 16S rRNA gene sequences can be found in bacteria with divergent genomes, the 21 Sph isolates were assessed for biochemical (API) and physiological characteristics (swimming and twitching) to uncover their phylogenetic coherency.^26^

**Table 1:** Phylogenetic affiliation and origin of the Sph isolates

Strain (clade)	Accession number^a^	Closest relative (% identity)	Closest cultivated relative (% identity)	Membrane type^b^	Feed water
Sph1 (A)	KP866793	*Sphingomonas* sp. GW5 (100%)	*Sphingomonas parapaucimobilis* strain NBRC 15100 (99%)	RO	Surface water (A.G. Wildervanckkanaal)
Sph2 (B)	KP866794	*Sphingomonas pseudosanguinis* strain HPLM-2 (100%)	*Sphingomonas sanguinis* strain NBRC 13937 (99%)	RO	Industrial wastewater (Starch production)
Sph3 (B)	KP866795	*Sphingomonas pseudosanguinis* strain HPLM-2 (99%)	*Sphingomonas sanguinis* strain NBRC 13937 (99%)	RO	Industrial wastewater (Starch production)
Sph4 (C)	KP866796	*Sphingobium* sp. EMB 221 (99%)	*Sphingobium yanoikuyae* strain NBRC 15102 (99%)	NF	Municipal wastewater
Sph5 (D)	KP866797	*Sphingomonas sanguinis* strain BAB-7166 (99%)	*Sphingomonas sanguinis* strain NBRC 13937 (99%)	MF	Tap water
Sph6 (E)	KP866798	*Sphingomonas echinoides* strain NRRL B-3127 (100%)	*Sphingomonas echinoides* strain NBRC 15742 (99%)	RO^c^	Tap water
Sph7 (E)	KP866799	*Sphingomonas echinoides* strain NRRL B-3127 (100%)	*Sphingomonas echinoides* strain NBRC 15742 (99%)	RO^c^	Tap water
Sph10 (F)	KP866800	*Sphingomonas* sp. ZJ116 (100%)	*Sphingomonas melonis* strain DAPP-PG 224 (100%)	RO	Industrial wastewater (Starch production)
Sph11 (G)	KP866801	*Sphingomonas* sp. V1-2 (99%)	*Sphingomonas hankookensis* strain ODN7 (99%)	RO	Industrial wastewater (Starch production)
Sph16 (H)	KP866802	Uncultured bacterium clone HK34-1-11-4 (100%)	*Sphingomonas aquatilis* strain NBRC 16722 (98%)	RO	Surface water (A.G. Wildervanckkanaal)
Sph19 (A)	KP866803	*Sphingomonas* sp. GW5 (99%)	*Sphingomonas parapaucimobilis* strain NBRC 15100 (99%)	RO	Surface water (A.G. Wildervanckkanaal)
Sph22 (I)	KP866804	Uncultured bacterium HOClCi53 (100%)	*Sphingomonas hankookensis* strain ODN7 (99%)	RO	Surface water (A.G. Wildervanckkanaal)
Sph25 (A)	KP866805	*Sphingomonas* sp. GW5 (100%)	*Sphingomonas parapaucimobilis* strain NBRC 15100 (99%)	RO	Surface water (A.G. Wildervanckkanaal)
Sph27 (J)	KP866806	*Sphingomonas* sp. 2R-2 (99%)	*Sphingomonas hankookensis* strain ODN7 (99%)	RO	Industrial wastewater (Starch production)
Sph29 (A)	KP866807	*Sphingomonas* sp. GW5 (99%)	*Sphingomonas parapaucimobilis* strain NBRC 15100 (99%)	RO	Industrial wastewater (Starch production)
Sph30 (A)	KP866808	*Sphingomonas* sp. GW5 (99%)	*Sphingomonas parapaucimobilis* strain NBRC 15100 (99%)	RO	Industrial wastewater (Starch production)
Sph31 (A)	KP866809	*Sphingomonas* sp. GW5 (100%)	*Sphingomonas parapaucimobilis* strain NBRC 15100 (99%)	RO	Industrial wastewater (Starch production)
Sph32 (A)	KP866810	*Sphingomonas* sp. GW5 (100%)	*Sphingomonas parapaucimobilis* strain NBRC 15100 (99%)	RO	Industrial wastewater (Starch production)
Sph33 (K)	KP866811	*Sphingopyxis macrogoltabida* strain BSN54 (100%)	*Sphingopyxis soli* strain BL03 (99%)	RO	Industrial wastewater (Starch production)
Sph46 (L)	KP866812	*Sphingomonas* sp. HX-H01 (99%)	*Sphingobium xenophagum* strain BN6 (99%)	NF	Anoxic groundwater
Sph57 (L)	KP866813	*Sphingomonas* sp. HX-H01 (99%)	*Sphingobium xenophagum* strain BN6 (99%)	NF	Anoxic groundwater

^a^The 16S rRNA sequences of the Sph isolates have been deposited in Genbank under the accession numbers KP866793 - KP866813

^b^RO = reverse osmosis, NF = nanofiltration, MF = microfiltration

^c^Bacteria isolated from membranes used in laboratory experiments

### Physiological and biochemical characteristics

The ability of the Sph isolates to proliferate at different temperatures, salinities, and pH values was assessed. Growth was assessed for two weeks on a daily basis and qualitatively via macroscopic observation. All isolates grew at a temperature range of 8 to 37 °C (Table 2). Sph16 and Sph57 grew between 8 and 42 °C, albeit at much lower growth rates compared to their optimum temperature. All Sph isolates grew at NaCl concentrations between 0 and 3.5%, and almost half (10/21) grew at 5.0% NaCl. All isolates grew between pH values of 5 to 9 and many were either able to grow at pH 4 or at pH 10 (Table 2). To compare the physiological features of the Sph isolates to closely related strains we made an inventory of *Sphingomonas* type strains (Supplementary Table 2; inclusion was based on alphabetical order). The biochemical properties of the 21 Sph isolates were profiled using API 20NE strips (Supplementary Table 3). To compare the characteristics of our Sph isolates with closely related strains, we made an inventory of *Sphingomonas* type strains (Supplementary Table 3: inclusion was based on alphabetical order). All Sph isolates were able to assimilate glucose, maltose, mannose and arabinose, except Sph6, which was unable to assimilate mannose. In addition, most Sph isolates tested positive for N-acetylglucosamine and malate assimilation, and for ß-galactosidase activity.

**Table 2:** Physiological characteristics of the Sph isolates

Strain (clade)	Swimming		Swarming	Twitching		Growth ranges		
	Micro^a^	Macro^b^		Micro^c^	Macro^d^	pH	Temp (°C)	Salt(NaCl% w/v)
Sph1 (A)	+	-	-	+	+	5.0–10.0	8–37	0–5.0
Sph2 (B)	+	+	-	+	+	5.0–10.0	8–37	0–5.0
Sph3 (B)	+	-	-	+	+	5.0–10.0	8–37	0–5.0
Sph4 (C)	+	+	-	+	-	5.0–10.0	8–37	0–3.5
Sph5 (D)	+	-	+	+	+	5.0–10.0	8–37	0–5.0
Sph6 (E)	+	+	+	+	+	5.0–10.0	8–37	0–5.0
Sph7 (E)	+	+	-	+	-	4.0–9.0	8–37	0–3.5
Sph10 (F)	-	+	-	+	-	5.0–10.0	8–37	0–3.5
Sph11 (G)	+	+	+	+	+	6.0–10.0	8–37	0–3.5
Sph16 (H)	-	-	-	+	+	4.0–10.0	8–42	0–3.5
Sph19 (A)	+	+	+	+	+	5.0–9.0	8–37	0–5.0
Sph22 (I)	+	+	-	+	+	5.0–10.0	8–37	0–3.5
Sph25 (A)	+	-	+	+	-	5.0–9.0	8–37	0–5.0
Sph27 (J)	+	+	-	+	+	5.0–9.0	8–37	0–3.5
Sph29 (A)	+	+	-	+	+	5.0–9.0	8–37	0–5.0
Sph30 (A)	+	+	+	+	+	5.0–10.0	8–37	0–5.0
Sph31 (A)	+	+	+	+	+	5.0–9.0	8–37	0–5.0
Sph32 (A)	+	+	+	+	+	5.0–10.0	8–37	0–5.0
Sph33 (K)	-	-	-	+	-	4.0–10.0	8–37	0–3.5
Sph46 (L)	+	-	-	+	+	5.0–10.0	8–37	0–3.5
Sph57 (L)	+	-	-	+	+	5.0–10.0	8–42	0–3.5

^a^Swimming was assayed microscopically by phase contrast microscopy

^b^Swimming and swarming was assayed macroscopically by plate assays

^c^Twitching was assayed microscopically by growing the strains on TMGG medium amidst of a microscopic slide and a glass coverslip

^d^Twitching was assayed macroscopically by growing the cells on twitching plates

### Twitching motility and pili

**Fig. 2:**
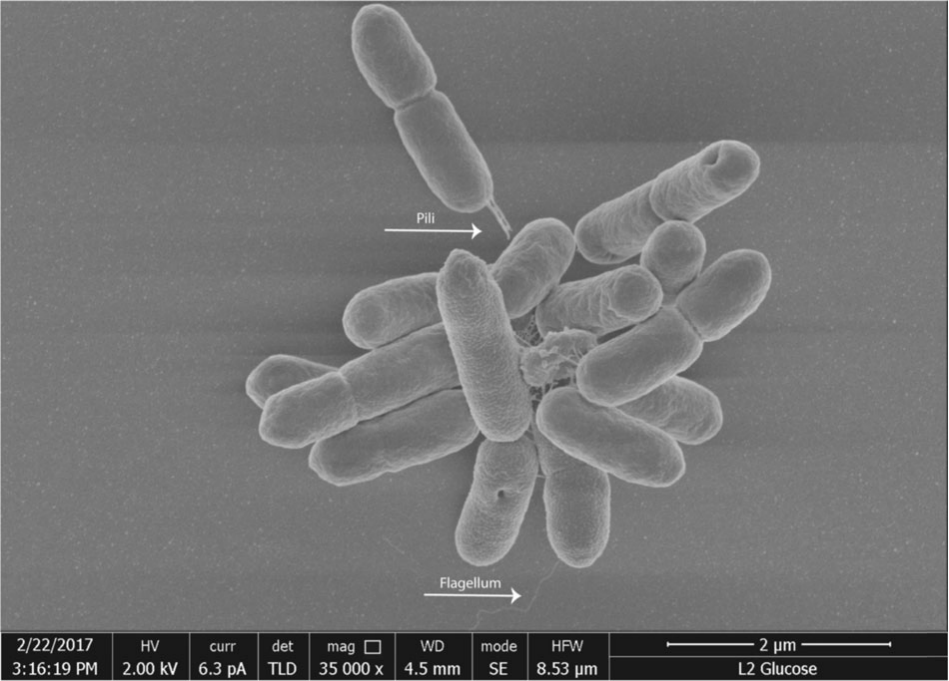
Scanning electron micrograph of membrane isolate Sph1 mounted on Poly–l–lysine coated coverslip

Twitching was assayed macroscopically using plate-based assays in which twitching is observed as the radial growth between the semisolid medium and the Petri dish. 17 of the 21 isolates (81%) twitched on the twitching plates, indicating that these Sph isolates possess active pili (Table 2). Twitching motility was also visualized microscopically by growing the Sph isolates on TMGG medium amidst of a microscopic slide and a glass coverslip (Supplementary Figure 1), which confirmed the macroscopic observations.^27^ All Sph isolates resembled the twitching phenotype of *Pseudomonas aeruginosa* strain PAK, which was observed using the same method.^27^ In line with these observations, pili-like structures were observed for Sph1 using SEM imaging (Fig. 2).

### Auto-aggregation and biofilm formation of 12 representative strains

**Fig. 3:**
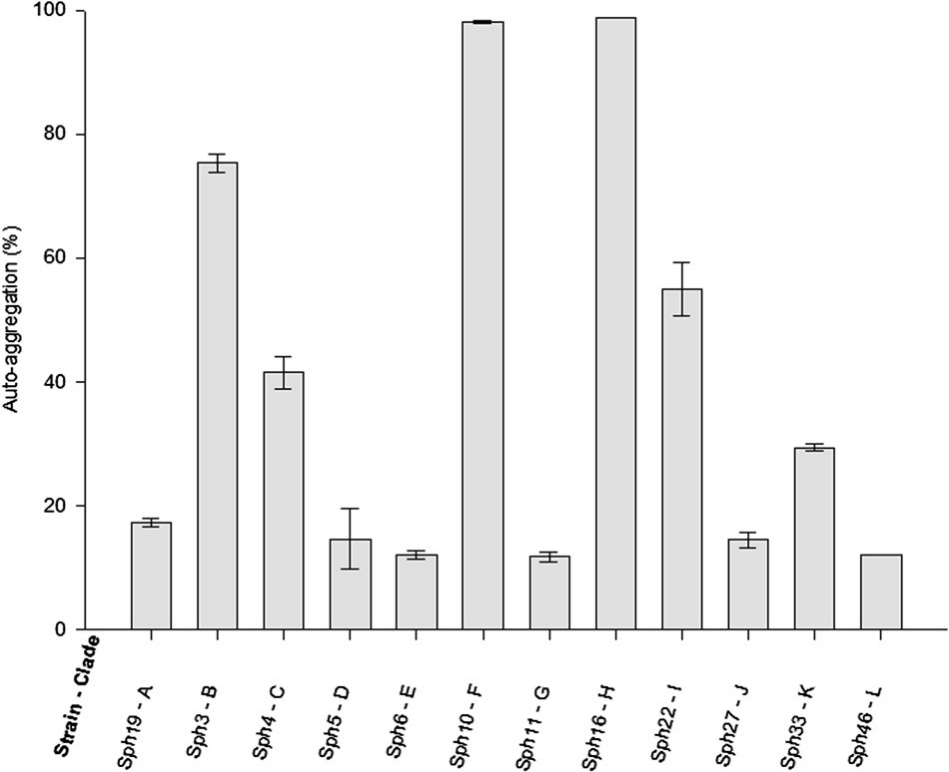
Auto-aggregation of the selected Sph isolates after 24 h of incubation

**Fig. 4:**
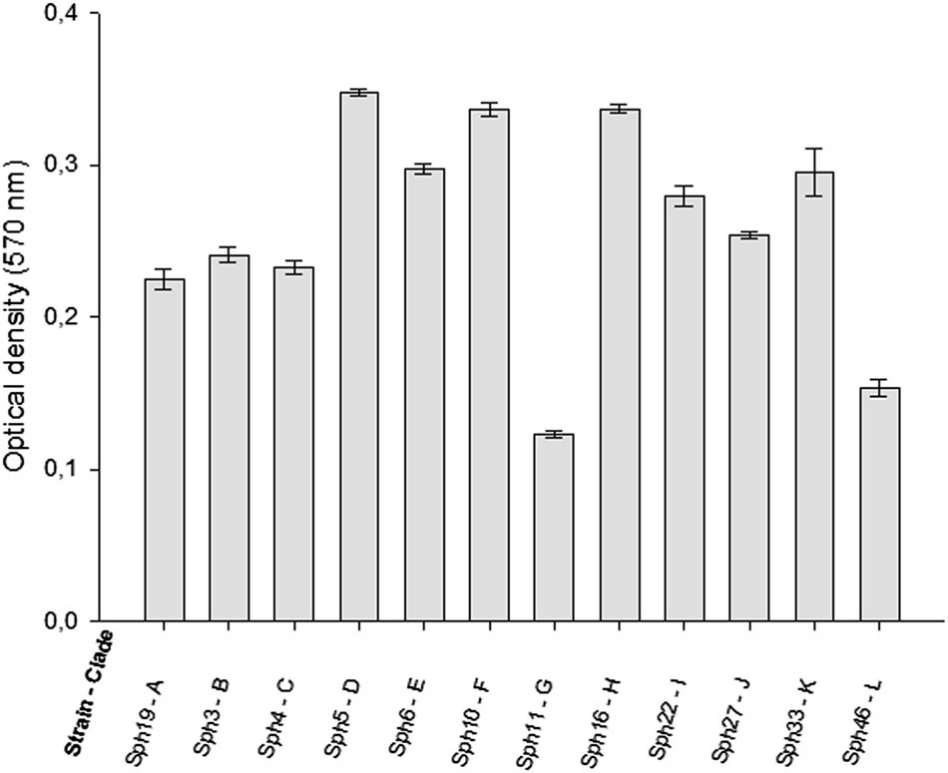
Biofilm formation of the selected Sph isolates: OD570 values of the crystal violet as measure of the amount of attached biomass after 16 h of incubation

Auto-aggregation was investigated by making repeated absorbance measurements for twelve unique strains (one strain was randomly selected per clade; Fig. 3). This observation was confirmed by microscopic observations. The ability to form a biofilm under static conditions was assessed using the microtiter plate assay for the 12 representative strains. All representative strains formed biofilms, although the amount of attached biomass differed between strains (Fig. 4). We were unable to determine whether the Sph isolates formed co-aggregates, because the Sph isolates cannot be differentiated by microscopic observation and the spectrophotometric method lacked resolution to differentiate between auto-aggregation and co-aggregation.

### Swimming motility and flagella

The ability of the Sph isolates to swim and swarm was investigated on plates solidified with agar using *P. aeruginosa* PAO1 as positive control. Although *P. aeruginosa* PAO1 tested positive, none of the Sph isolates was able to swim under these conditions. However, microscopic observations by phase contrast microscopy indicated that 18 Sph isolates (86%) are able to swim (Table 2). When agar was replaced with gellan gum, 14 Sph isolates (66%) tested positive for either swimming or swarming. SEM imaging revealed that Sph1 indeed produced a monotrichous polar flagellum (Fig. 2) and confirmed that besides clade A (Sph1), members of 8 of the other 10 clades produced a polar flagellum or monotrichous flagella (Supplementary Fig. 2).

## Discussion

This study characterizes the physiology of *Sphingomonadaceae* membrane isolates, members of a family that previous studies have shown to be dominant on fouled membranes.^17,22,23^ Other studies investigating the microbial diversity and function of bacteria on high-pressure membranes used molecular identification technologies or investigated a small number of bacterial species for which the relative abundancy was unknown.^6,11,15–18,25,28^ Comparison of the physiological features of the Sph isolates (Table 2) to those of *Sphingomonas* type strains (Supplementary Table 2) shows that the tolerance for different temperatures, salt concentrations and pH values is high for the Sph isolates.^22^ Particularly their ability to tolerate multiple stressors is not often found among *Sphingomonadaceae*. For instance, *S. dokdonensis and S. aestuarii* are able to tolerate 5.0% NaCl, but their growth range for different temperatures and pH values is more restricted compared to the Sph isolates (Table 2 and Supplementary Table 2). The results presented here therefore highlight that physiological traits that were hitherto not affiliated to biofouling do contribute to effective membrane colonization by *Sphingomonadaceae*.

The versatile physiology of the Sph isolates explains why they maintain themselves on membrane surfaces and in the membrane installations. During membrane filtration, salts and carbohydrates accumulate on the membrane.^29^ While the accumulated carbohydrates function as nutrient supply, the salt concentrations may become a stressor for microbial growth. Moreover, the pH is frequently and swiftly changed by the cleaning procedures that rely on acidic and alkaline cleaning agents to remove fouling.^30^ These observations, as suggested before, support the hypothesis that the conditions on the membrane surface are highly selective for the bacterial kingdom.^18^ This is also in line with the phylogenetic affiliation of the Sph isolates, which are most closely related to other *Sphingomonadaceae* isolated from water treatment systems or biofilms (Fig. 1).

However, the results presented here also clearly indicate that relative abundance, although used as an indicator of fitness of a whole group, would not have revealed the specific traits of the *Sphingomonadaceae* presented here. Conditions at the membrane surface are different from other habitats from which *Sphingomonadaceae* have been isolated. It is well-known that bacteria genetically and phenotypically adapt to changing environmental conditions, but this does not necessarily lead to sequence differences in the ribosomal operon (16S rRNA gene).^26^ This implies that although molecular identification technologies, such as next generation sequencing and FISH, accurately identify microbial communities, comparative analysis is limited by the reference database. Hence, culture-dependent approaches are key to discover the physiological and biochemical traits of representative bacteria. Some physiological features of the dominant bacteria on fouled membranes may have, for this reason, remained unknown. To determine which physiologies are required for membrane colonization, and to gain a better understanding of membrane biofouling, community studies should preferably be combined with culture-dependent or whole-genome analysis to uncover strain specific traits.

The number of different *Sphingomonadaceae* strains characterized in this study was limited to 21 strains because 39 of the membrane isolated strains did not belong to the *Sphingomonadaceae* family (Table 1 and Supplementary Table 1). These strains resisted the combination of streptomycin and piperacillin, although they do not belong to the *Sphingomonadaceae* family. However, this is not unpredictable based on the resistance of other bacteria to these antibiotics.^31^ Molecular identification is therefore essential in selecting *Sphingomonas* strains when using this selective isolation method. Some of the strains that were isolated from the same membrane share identical 16S rRNA sequences and appear not be unique but are rather paraphyletic because their physiological and biochemical traits are very similar. The strains Sph6 and Sph7 formed one clade, but differentiated in their biochemical and physiological behavior.

There are several hypotheses for the dominance of *Sphingomonadaceae* in membrane biofilm formation. These include: (I) oligotrophic growth, (II) the arrangement of their cell wall, which is hydrophobic due to the presence of glycosphingolipids rather than lipopolysaccharides, (III) the EPS composition which facilitates membrane adhesion and also provides strong rigidity, and (IV) surface motility by twitching and swimming.^28,32,33^ Only one study has investigated the behavior of bacteria isolated from the influent of high-pressure membranes and this study illustrates that most of the culturable bacteria present in the feed, including those belonging to the *Sphingomonadaceae* family, are nonmotile.^34^ Members of the *Sphingomonas* genus are known to be nonmotile or contain a single polar flagellum^21^ (Supplementary Table 2). All Sph isolates were motile, either by swimming, swarming or by twitching (Table 2). These observations support the notion that flagella or pili might provide an advantage for membrane attached *Sphingomonadaceae*. Accordingly, Pang and coworkers, who showed that a *Sphingomonas* membrane isolate possessed both twitching and swarming motility, suggested that surface motility might be important in mediating (membrane) surface colonization.^28^

Flagella and type IV pili have multiple functions during biofilm formation. In membrane filtration, two flow directions affect membrane adhesion and biofilm formation: the flow parallel to the membrane (i.e., cross-flow) and the flow perpendicular to the membrane (i.e., permeate drag force).^35^ Appendages like type IV pili and flagella are commonly used by bacteria to mediate surface attachment, but permeate drag forces make these appendages redundant for membrane adhesion.^35^ However, long-term biofouling experiments have shown that biofilm formation does not occur on the entire membrane, but strictly occurs close to the feed spacer where the cross-flow is quasi-stagnant.^36,37^ Collectively, these observations imply that bacterial adhesion must occur on the entire membrane, but that biofilm formation is impeded on most locations due to the high cross-flow. *Sphingomonas leidyi* (previously *Caulobacter leidyi*) produces, in a process in which both flagella and pili play a key role, a holdfast that acts as a strong surface-adhesin for *Caulobacter* species.^38,39^ Due to the high cross-flow velocities, this holdfast can be very favorable for the Sph isolates to establish a stable membrane interaction. Type IV pili (twitching) are important to form microcolonies via cell-aggregation.^40–43^ For *Sphingomonas natatoria*, pili-mediated cell-aggregation has been proven essential for the dominance of *S. natatoria* in dual-species biofilms with *Micrococcus luteus*.^43^

Like pili, flagella also have many important functions during the early and late biofilm formation stages. In the earliest biofilm stage, flagella provide a manner to sense the surface when flagellar rotation is interrupted. This mechano-sensing mechanism provides a signal to initiate biofilm formation.^44^ *Sphingomonadaceae* and other closely related bacteria profit particularly from a flagellum because their cell wall is hydrophobic due to the presence of glycosphingolipids.^45^ Because of the hydrophilicity of the flagellum and the hydrophobicity of the cell wall, the interaction between the surface and *Sphingomonadaceae* is elastic, which stimulates surface exploration.^45^ Flagella are produced in mature biofilms, particularly at the outer edge of the biofilm, which indicates that flagella can be used for biofilm expansion.^46^

EPS plays a pivotal role in membrane biofouling because it provides embedded bacteria protection against cleaning agents.^9,15^ All Sph isolates formed biofilms, but the amount of attached biomass after 16 h was different for most of the Sph isolates (Fig. 4). This is remarkable because EPS production is considered as an important feature for membrane colonization and survival.^9,16^ *Sphingomonadaceae* produce EPS with high mechanical and heat resistance; those produced by *S. paucimobilis* are even able to withstand autoclaving.^47^ Therefore EPS quality might be a more important feature than EPS quantity. We have also shown that the Sph isolates have a flexible metabolism, which is beneficial for survival under conditions of changing nutrient supplies (Supplementary Table 3).

Biofouling remains the most frequent observed membrane fouling type, for reasons described above. Improved strategies to prevent biofouling are highly demanded because the current strategies to prevent (pre-treatment of the influent) or control (membrane cleaning) biofouling are inadequate, relatively expensive, can damage the top layer of the membrane, lead to membrane downtime and add to the CO_2_ footprint.^30^ Different types of cleaning agents can be used for biofouling removal. Alkaline and acidic cleaning removes organic foulants on membranes and destroys the cell wall of microbes, respectively. Metal chelating agents and surfactants can be used to disintegrate EPS layers by removal of divalent cations and solubilization of macromolecules, respectively.^30^ In many cases, membrane cleaning loses its efficiency over time and this coincides with changes in the microbial community.^48^ We show that the Sph isolates are, as free-floating bacteria, capable to grow under pH values that approach those used to remove membrane biofouling. This is in line with the work of Bereschenko et al.,^14^ who showed that members of the *Sphingomonadaceae* family are able to persist membrane cleaning, but this is uncommon for the *Sphingomonadaceae* family as a whole.^21^ The results of this study therefore indicate that the conditions on the membrane surface are selective for microbial populations that withstand these conditions. As a consequence, the biofilm embedded cells and the EPS layer become more difficult to remove over time.

Knowledge on the efficiency of membrane cleaning agents and their effect on bacteria is limited, possibly because the manufacturers in most cases are not very willing to share details. However, the results shown here indicate that biofilm ageing is an important factor that should be taken into account when investigating the proficiency of membrane cleaning agents under representative conditions. Another implication would be to frequently change the cleaning strategy to prevent microbial adaptation. However, similar approaches have been studied before and with low efficiency.^30^ This might indicate that aged biofilms in general are difficult to remove. The aim of more effective strategies should, therefore, be twofold: (I) prevent biofilm formation on the membrane surface and (II) prevent biofilms from adapting to the conditions during membrane cleaning.

## Methods

### Enrichment and isolation

A total of 60 pure cultures were obtained from fouled membranes (Table 1 and Supplementary Table 1). Four membranes were acquired from four different full-scale water purification systems, and two membranes were obtained from laboratory experiments. The *Sphingomonadaceae* strains isolated in this study are listed in Table 1. *Pseudomonas aeruginosa* PAO1 (DSM 1707) was obtained from the Deutsche Sammlung von Mikroorganismen und Zellkulturen (DSMZ; Braunschweig, Germany). This bacterium was selected in this study because of its ability to swim, swarm and twitch and could therefore be used as positive control. *P. aeruginosa* PAO1 is a Gram-negative model strain for biofilm research in general and has been thoroughly used to investigate membrane biofouling.^49,50^ Unless stated otherwise, *P. aeruginosa* PAO1 and the *Sphingomonadaceae* isolates were grown in R2 broth (Teknova, York, UK) at 30 °C while shaken at 200 rpm.

For the enrichment of *Sphingomonadaceae*, biomass scraped from membranes was three times sonicated (40 kHz for 5 min) and vortexed (2 min), and plated on L9 minimal salt medium supplemented with streptomycin and piperacillin to select for *Sphingomonas* strains.^24^ Plates were incubated for three days at 31 °C, and selected colonies were re-streaked three times on R2 agar (Merck Millipore, Darmstadt, Germany) to obtain pure cultures. All isolates were stored at −80 °C using the Viabank™ (Medical Wire & Equipment, Corsham, Wiltshire, UK) cryoprotection system.

### Bacterial identification

Bacterial identification was performed using 16S rRNA gene sequencing. Genomic DNA was extracted from single colonies grown on R2 agar plates using the FastDNA® SPIN Kit for soil (Bio 101 Corp., Vista, CA) according to manufacturer’s instructions. The 16S rRNA gene was amplified using primers 7f (5′-GACGGATCCAGAGTTTGATYWTGGCTCAG-3′)^51^ and 1541r (5′-AAGGAGGTCATCCANCCRCA-3′).^52^ For isolates Sph4, Sph11 and Sph19 the primer set 7f/1541r was unsuccessful in delivering an amplicon, and instead the primer set 27f (5′-GTTTGATCCTGGCTCAG-3′) and 1492r (5′-CGGCTACCTTGTTACGAC-3′) was used.^53^ DNA amplification was carried out using a mixture (total volume, 50 μL) containing 2 μL of DNA extract, 1 U of *Taq* polymerase (Amersham Biosciences, Roosendaal, The Netherlands), 0.25 mM of deoxynucleoside triphosphates, 0.1 μM of each primer (Eurofins MWG Operon, Ebersberg, Germany), and 1 × PCR buffer under the following conditions: initial denaturation for 5 min at 94 °C, followed by 30 cycles of 30 s denaturation at 94 °C, 45 s annealing at 54 °C and 1.5 min elongation at 72 °C. Post-elongation was performed for 5 min at 72 °C. Amplicons were sequenced using the Sanger method using the same primers at (BaseClear BV, Leiden, The Netherlands).

### Phylogenetic analysis

For all the membrane isolates, the 16S rRNA gene was sequenced as described above. The forward and reverse sequences were assembled into contiguous reads and corrected with ChromasPro software (Technelysium Pty Ltd., Brisbane, Australia). After assembly, DECIPHER was used to check for chimeras.^54^ Sequences were aligned using SINA Alignment Service (V1.2.11).^55^ The aligned almost full-length 16S rRNA sequences were merged with the SSU Ref NR 99 128 database (SSU Ref NR 128, September 2016) and a phylogenetic tree was constructed using the ARB software package (version arb-6.0.1).^56^ The phylogenetic tree was calculated using the ARB neighbor-joining algorithm from 1000 bootstraps samples with Jukes–Cantor correction and terminal filtering.

### Biochemical tests

Biochemical properties of 21 selected isolates were determined using API 20NE strips according to manufacturer’s instruction (BioMerieux, La Balme-les- Grottes, France). All tests were performed in duplicate and the results were interpreted following the manufacturer’s instruction.

### Motility assays

Swimming, swarming, and twitching motility of the Sph isolates was assayed macroscopically and microscopically, in duplicate.^27^ To assay swimming and swarming motility macroscopically, an overnight grown R2 broth culture was inoculated to an OD_600_ of 0.1 in fresh R2 medium, grown to mid-exponential phase and centrally inoculated on M8 medium containing per litre 12.8 g Na_2_HPO_4_ × 7H_2_O, supplemented with 3.0 g agar or 3.0 g gellan gum (Wako pure chemical industries, Neuss, Germany), 10 mL of 20% (w/v) glucose, 25 mL of 20% (w/v) casamino acids, and 1 mL of 1 M MgSO_4_, and grown for 5 days.^27^ To assay swimming microscopically, a mid-exponential culture was observed using phase contrast microscopy (Leica DM 750, Heerbrugg, Switzerland). To assay twitching macroscopically, colonies that were grown overnight on 1.5% LBA (containing per litre 4.0 g Tryptone, BD Difco, Breda, The Netherlands), 2.0 g yeast extract (Merck Millipore, Darmstadt, Germany), and 2.0 g NaCl were picked and point inoculated to the bottom of LBA plates containing 1.0% agar, and incubated at 30° C for 3 days. To assay twitching microscopically, overnight grown colonies on twitching motility gellan gum plates (TMGG) (containing per 100 mL: 0.8 g gellan gum, 0.4 g tryptone, 0.2 g yeast extract, 0.2 g NaCl, 0,1 g MgSO_4_ 7H_2_O, were picked using a sterile plastic inoculation loop and streaked on a thin layer of a TMGG coated microscopic slide, covered by a glass coverslip and incubated at 30 °C. Microscopic images were recorded every 24 h for 3 days using phase contrast microscopy equipped with a camera (Leica MC 120 HD) and connected to the LAS 4.5 software.

### Growth parameters

To test growth at different pH values, NaCl concentrations, and temperatures, an overnight grown culture was used to inoculate R2 broth to an OD_600_ of 0.1, and grown with the parameters specified below. To test growth at different pH values, the pH of R2 broth was set to 3.5, 4.0, 4.5, 5.0, 5.5, and 6.0 using a citric acid (0.5 M)—disodium hydrogen phosphate (0,5 M) buffered solution, and to pH 8.0, 8.5, 9.0, 9.5, 10.0, and 10.5 using a sodium carbonate (0.5 M)—sodium bicarbonate (0.5 M) solution. To test growth at different NaCl concentrations, NaCl was added to R2 broth to 0, 3.5, and 5.0% (w/v). Growth was tested at temperatures of 8, 15, 30, 37, 40, 42, and 45 °C. Determination of growth parameters was performed in duplicate and growth was monitored after two weeks by eye. The cultures were given a negative score for no visual turbidity compared to the inoculated medium without carbon source and no visual turbidity compared to blanc medium without inoculum.

### Electron microscopy

For scanning electron microscopy (SEM) imaging, cells were grown in R2 broth at 30 °C and shaking at 200 rpm, and harvested in the mid-exponential phase. Bacterial cells were mounted on coverslips coated with poly–l–lysine (Fisher Scientific, Landsmeer, The Netherlands) and fixed with 3% (v/v) glutaraldehyde and 1% (v/v) OsO_4_, respectively. The sample was fixed for 1 h at room temperature, dehydrated in graded ethanol solutions in water (30, 50, 70, 80, 90, 96, and 100%) for 15 min each, and critical point dried using liquid carbon dioxide as transition fluid. The coverslips were coated with tungsten and examined with a scanning electron microscope (FEI Magellan 400, Eindhoven, The Netherlands).

### Biofilm formation under static conditions

Biofilm formation of the Sph isolates was assayed under static conditions using a microtiter plate assay as described previously, with some modifications.^27^ The strains were grown overnight, diluted in fresh R2 broth medium to an OD_600_ of 0.1 and grown to mid-exponential phase. Three wells of a polystyrene 96-wells flat-bottomed, hydrophobic polystyrene microtiter plate (Corning incorporated, New York) were inoculated with 100 μL of the mid-exponential phase culture (OD_600_ of 0.1) and statically incubated at 30 °C. Wells containing 100 μL R2 broth were taken as negative control. After 16 h, the liquid was removed and wells were washed twice with sterile milliQ water. The plates were air-dried and the attached biomass was stained for 10 min with 125 μL 0.1% (w/v) crystal violet. The unbound crystal violet was removed by rinsing the plates two times with milliQ water, after which the plates were air-dried. Attached biomass was subsequently solubilized in 150 μL 70% ethanol. The optical density of this solution was measured at 570 nm using a microtiter plate reader (Victor 3-1420 Multilabel Counter, Perkin-Elmer, Waltham, MA, USA). The assay was performed in triplicate and the results were averaged.

### Auto-aggregation

The ability to form cell aggregates was assayed quantitatively using OD_600_ measurements as follows.^57^ The cell suspensions (grown for 24 h in R2 medium) were centrifuged at 5000 × *g* for 15 min at 4 °C and washed twice using buffered KCl (pH 6.0; 50 mM KCl, 1 mM CaCl_2_, 1 mM KH_2_PO_4_, 0,1 mM MgCl_2_). The turbidity of this culture was adjusted to OD_600_ of 1.0 and an aliquot of 1 mL of this solution was pipetted into a micro-cuvette (VWR, Leuven, Belgium). The OD_600_ was measured immediately (OD(0)) and after 24 h (OD(24)). The percentage of auto-aggregation after 24 h was calculated by Eq. (1):

1 $${\mathrm{\% }}\,{\mathrm{of}}\,{\mathrm{aggregation}} = \frac{{{\mathrm{OD}}(0) - {\mathrm{OD}}(24)}}{{{\mathrm{OD}}(0)}} \cdot 100$$

## Supplementary information

SUPPLEMENTAL MATERIAL

## Data Availability

Data generated and analyzed during this study, including accession codes of deposited nucleotide sequences (KP866793 to KP866813) that are deposited in the Genbank are included in this published article and its Supplemental information file. Additional details available upon reasonable request.

## Supplementary information

**Supplementary information** accompanies the paper on the *npj Biofilms and Microbiomes* website (10.1038/s41522-018-0074-1).
