# Supplementary material for: Isolation and characterization of Sphingomonadaceae from fouled membranes
Source: NPJ Biofilms Microbiomes. 2019 Jan 25;5:6. doi: 10.1038/s41522-018-0074-1 (PMC6347639; doi:10.1038/s41522-018-0074-1)
Supplement: Supplementary file 1 — SUPPLEMENTAL MATERIAL [file 41522_2018_74_MOESM1_ESM.pdf]

Supplementary files

Isolation and characterization of *Sphingomonadaceae* from fouled membranes

Hendrik J. de Vries[1,2 *], Florian Beyer[1,2 *], Monika Jarzembowska[1], Joanna Lipińska [2], Paula van den Brink[2], Arie Zwijnenburg[2], Peer H.A. Timmers[1,2], Alfons J.M. Stams[1] and Caroline M. Plugge[1,2]

[*] Hendrik J. de Vries and Florian Beyer contributed equally to this work.

[1] Laboratory of Microbiology, Wageningen University & Research, Stippeneng 4, 6708 WE Wageningen, The Netherlands

[2] Wetsus, European Centre of Excellence for Sustainable Water Technology, Oostergoweg 9, 8911 MA Leeuwarden, The Netherlands

DESCRIPTION OF THE ISOLATION SOURCES

Isolation source A - Kisuma II: The isolates Sph16 and Sph22 were isolated from an reverse osmosis (RO) installation extensively reported in literature for its biofouling problems [1]. The installation treats surface water for process water production. The feed water is first pre-treated by coagulation, flocculation and sand filtration and ultrafiltration, before entering the two-stage RO system [1]. In this installation biofouling causes strong normalized pressure drop increase leading to a high cleaning frequency of about once per week.

Isolation source B - DWP Sas van Gent: The isolates Sph2, Sph3, Sph10, Sph11, Sph27 and Sph33 were retrieved from two lead RO membrane elements from an installation producing demineralised water from industrial wastewater effluent from a starch producing plant [2]. The isolates Sph2 and Sph3 were recovered from a membrane element that was taken out shortly after chemical cleaning of the RO system, while the isolates Sph10, Sph12, Sph27 and Sph33 were recovered from a membrane element that was not cleaned for several weeks prior to membrane autopsies [3]. The RO feed water is characterized by high temperatures (25 - 35 °C) and high concentration of total organic carbon (TOC; 15 – 25 mg TOC L^{-1}). The treatment consists of an inline flocculation with iron, dual media filtration, ultrafiltration, antiscalant dosing, first stage RO system, degasifiers and a second stage RO system [2]. When the effluent water quality is out of specifications, the RO is operated on drinking water to maintain demineralized water production. The RO stages are cleaned preventive once every 6 weeks.

Isolation source C - Engelse werk WTP: The isolates Sph46 and Sph57 were retrieved from a lead nanofiltration (NF) membrane element from an installation producing drinking water from anoxic groundwater, similar to the anoxic groundwater treating NF installations described in detail in [4,5]. The treatment main objectives is the removal of hardness, taste and organic micropollutants. The operation and performance of such anoxic groundwater treating NF plants is typically very stable and unproblematic, resulting in a cleaning frequency of once per year or less [5].

Isolation source D – Laboratory cleaning experiments of full-scale membrane samples: The isolates Sph1, Sph19, Sph25, Sph29, Sph30, Sph31 and Sph32 were retrieved from full-scale membrane samples after cleaning in a laboratory flow-cell setup [3]. Sph1, Sph19 and Sph25 membrane were retrieved from the membrane from isolation source A after laboratory cleaning, while Sph29, Sph30, Sph31 and Sph32 were retrieved from the laboratory cleaned membrane from isolation source B. For the cleaning experiments, smaller sheets of and spacer material from the full-scale 8” SWRO membrane elements (Isolation source A & B) were transferred to a membrane flow-cell [3] . The membranes were then cleaned using procedures similar to full-scale. A typical cleaning protocol included a) Pre-Rinse with demineralized water, b) Acid cleaning circulation with P3-ultrasil 73 (45 min; T = 45°C), c) Rinse with demineralized water, d) Alkaline cleaning circulation with P3-ultrasil 53 (90 min; T=37°C; %1.5; pH=9,6-10,0), e) Alkaline cleaning soaking with P3-ultrasil 53 Alkaline - P3-ultrasil 53 (30min; T=30-45°C), f) Rinse with demineralized water g) Sanitizing circulation with P3-oxonia active (60 min; T=max 25°C; %1.0), h) Final rinse with demineralized water. The cleanings were

performed at low pressure (~1bar) and high velocity (>0.2m s^{-1}), similar to typical full-scale cleaning procedures. To evaluate cleaning efficiencies in terms of operation (e.g. differential pressure drop and flux), the membrane and spacer sheets were operated in a high pressure laboratory setup before and after cleaning (~1h of operation). Only after the performance evaluation the flow-cells were opened for membrane autopsies and the *Sphingomonadaceae* were isolated. It is possible that the isolated *Sphingomonadaceae* isolates from these samples survived the cleaning. However, it is more likely that the *Sphingomonadaceae* isolates originate from the Wetsus feed water used for membrane performance evaluation after cleaning.

Isolation source E - MBR-NF: Isolate Sph4 was recovered from a 2.5" DOW NF270 membrane element operated on MBR effluent from an integrated MBR NF system for municipal wastewater treatment with 100% NF concentrate recirculation back to the MBR [6]. The NF feed was characterized by high concentration of total organic carbon (17±4 mg TOC L^{-1}), phosphorus (18±4 mg PO_4^{3-} L^{-1}) and sulfate (43±25 mg SO_4^{2-} L^{-1}) [6].

Isolation source F - NADIR MF: The isolate Sph5 was recovered from a Nadir MP005 microfiltration flat sheet membrane used for laboratory flow-cell biofouling studies. The flow-cells are operated on drinking water with additional dosage of easily biodegradable nutrients [7].

Isolation source G – ESPA2 RO: The isolates Sph6 and Sph7 were recovered from an accelerated laboratory flow-cell biofouling experiment and the flow-cell used was identical to the flow-cell used by Bereschenko et al., 2010 [8].

References

1. Bereschenko, L., et al., Molecular characterization of the bacterial communities in the different compartments of a full-scale reverse-osmosis water purification plant. *Applied and environmental microbiology*, 2008. **74**(17): p. 5297-5304.

2. Van den Broek, W., et al., Prevention of Biofouling in Industrial RO Systems: Experiences with Peracetic Acid. *Water Practice and Technology*, 2010. **5**(2): p. wpt2010042.

3. Beyer, F et al.,. Membrane fouling and chemical cleaning in three full-scale reverse osmosis plants producing demineralized water. J Engineer https://doi.org/10.1155/2017/6356751

4. van der Meer, W.G., et al., Optiflux®: from innovation to realisation. *Desalination*, 2003. **157**(1): p. 159-165.

5. Beyer, F., et al., Long-term performance and fouling analysis of full-scale direct nanofiltration (NF) installations treating anoxic groundwater. *Journal of Membrane Science*, 2014. **468**: p. 339-348.

6. Kappel, C., et al., Impacts of NF concentrate recirculation on membrane performance in an integrated MBR and NF membrane process for wastewater treatment. *Journal of Membrane Science*, 2014. **453**: p. 359-368

7. Dreszer, C., et al., In-situ biofilm characterization in membrane systems using Optical Coherence Tomography: Formation, structure, detachment and impact of flux change. *Water research*, 2014. **67**: p. 243-254.

8. Bereschenko, L. A., Stams, A.J.M., Euverink, G.J.W. & Van Loosdrecht, MC.M. Biofilm formation on reverse osmosis membranes is initiated and dominated by *Sphingomonas* spp. *Applied and environmental microbiology* **76**, 2623-2632 (2010).

Table S1: overview of the non-*Sphingomonadaceae* strains and their most closely related species.

Strain	Membrane	Feed water	Closest relative	Phylum
Sph8	RO	Tap water	*Pedobacter nutrimenti*	Bacteroidetes
Sph9	RO	Surface water	*Staphylococcus petrasii*	Firmicutes
Sph12	RO	Surface water	*Microbacterium hominis*	Actinobacteria
Sph13	RO	Surface water	*Pedobacter koreensis*	Bacteroidetes
Sph14	RO	Surface water	*Pedobacter koreensis*	Bacteroidetes
Sph15	RO	Surface water	*Pedobacter koreensis*	Bacteroidetes
Sph17	RO	Surface water	*Brevundimonas vesicularis*	Proteobacteria
Sph18	RO	Surface water	*Brevundimonas vesicularis*	Proteobacteria
Sph20	RO	Surface water	*Brevundimonas vesicularis*	Proteobacteria
Sph21	RO	Surface water	*Brevundimonas vesicularis*	Proteobacteria
Sph23	RO	Surface water	*Pedobacter koreensis*	Bacteroidetes
Sph24	RO	Surface water	*Pedobacter koreensis*	Bacteroidetes
Sph26	RO	Surface water	*Massilia aurea*	Proteobacteria
Sph28	RO	Surface water	*Brevundimonas vesicularis*	Proteobacteria
Sph34	RO	Surface water	*Pedobacter koreensis*	Proteobacteria
Sph35	RO	Surface water	*Microbacterium saccharophilum*	Actinobacteria
Sph36	RO	Surface water	*Pedobacter steynii*	Bacteroidetes
Sph37	RO	Surface water	*Pedobacter koreensis*	Bacteroidetes
Sph38	RO	Surface water	*Micrococcus antarcticus*	Actinobacteria
Sph39	RO	Surface water	*Chryseobacterium taeanense*	Bacteroidetes
Sph40	NF	Anoxic Groundwater	*Microbacterium oxydans*	Actinobacteria
Sph41	NF	Anoxic Groundwater	*Microbacterium oxydans*	Actinobacteria
Sph42	NF	Anoxic Groundwater	*Rhodococcus cerastii*	Actinobacteria
Sph43	NF	Anoxic Groundwater	*Rhodococcus cerastii*	Actinobacteria
Sph44	NF	Anoxic Groundwater	*Rhodococcus cerastii*	Actinobacteria
Sph45	NF	Anoxic Groundwater	*Dyella ginsengisoli*	Proteobacteria
Sph47	NF	Anoxic Groundwater	*Dietzia cercidiphylli*	Actinobacteria
Sph48	NF	Anoxic Groundwater	*Dyella ginsengisoli*	Proteobacteria
Sph49	NF	Anoxic Groundwater	*Dyella ginsengisoli*	Proteobacteria
Sph50	NF	Anoxic Groundwater	*Mitsuaria chitosanitabida*	Proteobacteria
Sph51	NF	Anoxic Groundwater	*Microbacterium oxydans*	Actinobacteria
Sph52	NF	Anoxic Groundwater	*Rhodococcus cerastii*	Actinobacteria
Sph53	NF	Anoxic Groundwater	*Microbacterium phyllosphaerae*	Actinobacteria
Sph54	NF	Anoxic Groundwater	*Rhodococcus cerastii*	Actinobacteria
Sph55	NF	Anoxic Groundwater	*Mitsuaria chitosanitabida*	Proteobacteria
Sph56	RO	Surface water	*Rhodococcus cerastii*	Actinobacteria
Sph58	NF	Anoxic Groundwater	*Microbacterium oxydans*	Actinobacteria
Sph59	NF	Anoxic Groundwater	*Microbacterium oxydans*	Actinobacteria
Sph60	RO	Surface water	*Terrabacter terrae*	Actinobacteria

Table S2. Physiological characteristics of *Sphingomonas* type strains.

Strain	Source	Motile by flagella	Growth tolerance			Reference
			pH	Temp (˚C)	Salt (NaCl % w/v)	
S. alpina	Soil	Yes	6.0–10.0	1–30	0–1.0	1
S. arantia		No	6.0–9.0	4–35	0–0.5	2
S. changbaiensis		Yes	5.0–8.0	20–33	0–0.1	3
S. desiccabilis		No	N.D.	15–37	0–4.0	4
S. dokdonensis		Yes	5.0–9.5	10–34	0–5.0	5
S. flava		No	6.5–8.5	18–30	0–2.5	6
S. formosensis		Yes	5.0–9.0	25–37	0–3.0	7
S. alaskensis	Freshwater	Yes	N.D.	4–48	0-3.0	8
S. astaxanthinifaciens		Yes	5.5–11.0	20–45	>0.25	9
S. daechungensis		No	5.0–9.0	15–37	0.5–3.0	10
S. fonticola		Yes	5.0–8.0	15–37	0–1.0	11
S. hankookensis		No	4.0–10.0	4–37	0–1.0	12
S. hengshuiensis		Yes	5.0–10.0	20–35	0–1.0	13
S. jaspsi		Yes	6.0–9.0	20.0–40.0	>0.25	14
S. abaci	Hospital table	No	N.D.	15–37	0–1.0	15
S.aerophila	Air	Yes	6.0–9.0	4–37	0–1.0	16
S. aestuarii	Sediment	No	N.D.	20–35	0-5.0	17
S. endophytica	Plant	Yes	N.D.	12–45	0–1.0	18
S. gei	Plant	Yes	6.0–9.0	4–33	0–2.0	19
S. gimensis	Mine	Yes	6.0–8.0	15–32	0–4.0	20
S. ginsengisoli	Ginseng field	No	N.D.	15–37	0-1.0	21

N.D.: not determined.

References to Table S2

1. Margesin, R., Zhang, D.-C. & Busse, H.-J. *Sphingomonas alpina* sp. nov., a psychrophilic bacterium isolated from alpine soil. *International journal of systematic and evolutionary microbiology* **62**, 1558-1563 (2012).

2. Jia, L. et al. *Sphingomonas arantia* sp. nov., isolated from Hoh Xil basin, China. *Antonie van Leeuwenhoek* **108**, 1341-1347 (2015).

3. Zhang, J.-Y., Liu, X.-Y. & Liu, S.-J. *Sphingomonas changbaiensis* sp. nov., isolated from forest soil. *International journal of systematic and evolutionary microbiology* **60**, 790-795 (2010).

4. Reddy, G. & Garcia-Pichel, F. *Sphingomonas mucosissima* sp. nov. and *Sphingomonas desiccabilis* sp. nov., from biological soil crusts in the Colorado Plateau, USA. *International journal of systematic and evolutionary microbiology* **57**, 1028-1034 (2007).

5. Yoon, J.-H., Lee, M.-H., Kang, S.-J., Lee, S.-Y. & Oh, T.-K. *Sphingomonas dokdonensis* sp. nov., isolated from soil. *International journal of systematic and evolutionary microbiology* **56**, 2165-2169 (2006).

6. Du, J. et al. *Sphingomonas flavus* sp. nov. isolated from road soil. *Archives of microbiology* **197**, 883-888 (2015).

7. Lin, S.-Y. et al. *Sphingomonas formosensis* sp. nov., a polycyclic aromatic hydrocarbon-degrading bacterium isolated from agricultural soil. *International journal of systematic and evolutionary microbiology* **62**, 1581-1586 (2012).

8. Vancanneyt, M. et al. *Sphingomonas alaskensis* sp. nov., a dominant bacterium from a marine oligotrophic environment. *International journal of systematic and evolutionary microbiology* **51**, 73-79 (2001).

9. Asker, D., Beppu, T. & Ueda, K. *Sphingomonas astaxanthinifaciens* sp. nov., a novel astaxanthin-producing bacterium of the family *Sphingomonadaceae* isolated from Misasa, Tottori, Japan. *FEMS microbiology letters* **273**, 140-148 (2007).

10. Huy, H. et al. *Sphingomonas daechungensis* sp. nov., isolated from sediment of a eutrophic reservoir. *International journal of systematic and evolutionary microbiology* **64**, 1412-1418 (2014).

11. Sheu, S.-Y., Chen, Y.-L. & Chen, W.-M. *Sphingomonas fonticola* sp. nov., isolated from spring water. *International journal of systematic and evolutionary microbiology* **65**, 4495-4502 (2015).

12. Yoon, J.-H., Park, S., Kang, S.-J., Kim, W. & Oh, T.-K. *Sphingomonas hankookensis* sp. nov., isolated from wastewater. *International journal of systematic and evolutionary microbiology* **59**, 2788-2793 (2009).

13. Wei, S. et al. *Sphingomonas hengshuiensis* sp. nov., isolated from lake wetland. *International journal of systematic and evolutionary microbiology* **65**, 4644-4649 (2015).

14. Asker, D., Beppu, T. & Ueda, K. *Sphingomonas jaspsi* sp. nov., a novel carotenoid-producing bacterium isolated from Misasa, Tottori, Japan. *International journal of systematic and evolutionary microbiology* **57**, 1435-1441 (2007).

15. Busse, H.-J., Hauser, E. & Kämpfer, P. Description of two novel species, *Sphingomonas abaci* sp. nov. and *Sphingomonas panni* sp. nov. *International journal of systematic and evolutionary microbiology* **55**, 2565-2569 (2005).

16. Kim, S.-J. et al. *Sphingomonas aerophila* sp. nov. and Sphingomonas naasensis sp. nov., isolated from air and soil, respectively. *International journal of systematic and evolutionary microbiology* **64**, 926-932 (2014).

17. Roh, S. W. et al. *Sphingomonas aestuarii* sp. nov., isolated from tidal flat sediment. *International journal of systematic and evolutionary microbiology* **59**, 1359-1363 (2009).

18. Huang, H.-Y. et al. *Sphingomonas endophytica* sp. nov., isolated from Artemisia annua L. *International journal of systematic and evolutionary microbiology* **62**, 1576-1580 (2012).

19. Zhu, L. et al. *Sphingomonas gei* sp. nov., isolated from roots of Geum aleppicum. *International journal of systematic and evolutionary microbiology* **65**, 1160-1166 (2015).

20. Feng, G.-D. et al. *Sphingomonas gimensis* sp. nov., a novel Gram-negative bacterium isolated from abandoned lead–zinc ore mine. *Antonie van Leeuwenhoek* **105**, 1091-1097 (2014).

21. An, D.-S. et al. *Sphingomonas ginsengisoli* sp. nov. and Sphingomonas sediminicola sp. nov. International journal of systematic and evolutionary microbiology 63, 496-501 (2013).

Table S3. Biochemical characteristics of the Sph isolates and the *Sphingomonas* type strains.

Species/Strain	Reference	Origin	Nitrate reduction	Indole production	glucose fermentation	arginine dihydrolase activity	urease activity	hydrolysis of esculin	hydrolysis of gelatin	ß-galactosidase activity	cytochrome oxidase	Assimilation											
												glucose	arabinose	mannose	mannitol	N-acetylglucosamine	maltose	gluconate	caprate	adipate	malate	trisodium citrate	phenylacetic acid
Sph1 - A																							
Sph2 - B																							
Sph3 - B																							
Sph4 - C																							
Sph5 - D																							
Sph6 - E																							
Sph7 - E																							
Sph10 - F																							
Sph11 - G																							
Sph16 - H																							
Sph19 - A																							
Sph22 - I																							
Sph25 - A																							
Sph27 - J																							
Sph29 - A																							
Sph30 - A																							
Sph31 - A																							
Sph32 - A																							
Sph33 - K																							
Sph46 - L																							
Sph57 - L																							
S. alpina	1	Soil																					
S. arantia	2																						
S. changbaiensis	3																						
S. desiccabilis	4																						
S. dokdonensis	5																						
S. flava	6																						
S. formosensis	7																						
S. alaskensis	8	Fresh water																					
S. astaxanthinifaciens	9																						
S. daechungensis	10																						
S. fonticola	11																						
S. hankookensis	12																						

S. hengshuiensis	13	
S. jaspsi	14	
S. abaci	15	Other
S.aerophila	16	
S. aestuarii	17	
S. endophytica	18	
S. gei	19	
S. gimensis	20	
S. ginsengisoli	21	

Green denotes a positive reaction, red a negative reaction and black denotes not determined.

References to Table S3

1. Margesin, R., Zhang, D.-C. & Busse, H.-J. *Sphingomonas alpina* sp. nov., a psychrophilic bacterium isolated from alpine soil. *International journal of systematic and evolutionary microbiology* **62**, 1558-1563 (2012).

2. Jia, L. et al. *Sphingomonas arantia* sp. nov., isolated from Hoh Xil basin, China. *Antonie van Leeuwenhoek* **108**, 1341-1347 (2015).

3. Zhang, J.-Y., Liu, X.-Y. & Liu, S.-J. *Sphingomonas changbaiensis* sp. nov., isolated from forest soil. *International journal of systematic and evolutionary microbiology* **60**, 790-795 (2010).

4. Reddy, G. & Garcia-Pichel, F. *Sphingomonas mucosissima* sp. nov. and *Sphingomonas desiccabilis* sp. nov., from biological soil crusts in the Colorado Plateau, USA. *International journal of systematic and evolutionary microbiology* **57**, 1028-1034 (2007).

5. Yoon, J.-H., Lee, M.-H., Kang, S.-J., Lee, S.-Y. & Oh, T.-K. *Sphingomonas dokdonensis* sp. nov., isolated from soil. *International journal of systematic and evolutionary microbiology* **56**, 2165-2169 (2006).

6. Du, J. et al. *Sphingomonas flavus* sp. nov. isolated from road soil. *Archives of microbiology* **197**, 883-888 (2015).

7. Lin, S.-Y. et al. *Sphingomonas formosensis* sp. nov., a polycyclic aromatic hydrocarbon-degrading bacterium isolated from agricultural soil. *International journal of systematic and evolutionary microbiology* **62**, 1581-1586 (2012).

8. Vancanneyt, M. et al. *Sphingomonas alaskensis* sp. nov., a dominant bacterium from a marine oligotrophic environment. *International journal of systematic and evolutionary microbiology* **51**, 73-79 (2001).

9. Asker, D., Beppu, T. & Ueda, K. *Sphingomonas astaxanthinifaciens* sp. nov., a novel astaxanthin-producing bacterium of the family *Sphingomonadaceae* isolated from Misasa, Tottori, Japan. *FEMS microbiology letters* **273**, 140-148 (2007).

10. Huy, H. et al. *Sphingomonas daechungensis* sp. nov., isolated from sediment of a eutrophic reservoir. *International journal of systematic and evolutionary microbiology* **64**, 1412-1418 (2014).

11. Sheu, S.-Y., Chen, Y.-L. & Chen, W.-M. *Sphingomonas fonticola* sp. nov., isolated from spring water. *International journal of systematic and evolutionary microbiology* **65**, 4495-4502 (2015).

12. Yoon, J.-H., Park, S., Kang, S.-J., Kim, W. & Oh, T.-K. *Sphingomonas hankookensis* sp. nov., isolated from wastewater. *International journal of systematic and evolutionary microbiology* **59**, 2788-2793 (2009).

13. Wei, S. et al. *Sphingomonas hengshuiensis* sp. nov., isolated from lake wetland. *International journal of systematic and evolutionary microbiology* **65**, 4644-4649 (2015).

14. Asker, D., Beppu, T. & Ueda, K. *Sphingomonas jaspsi* sp. nov., a novel carotenoid-producing bacterium isolated from Misasa, Tottori, Japan. *International journal of systematic and evolutionary microbiology* **57**, 1435-1441 (2007).

15. Busse, H.-J., Hauser, E. & Kämpfer, P. Description of two novel species, *Sphingomonas abaci* sp. nov. and *Sphingomonas panni* sp. nov. *International journal of systematic and evolutionary microbiology* **55**, 2565-2569 (2005).

16. Kim, S.-J. et al. *Sphingomonas aerophila* sp. nov. and Sphingomonas naasensis sp. nov., isolated from air and soil, respectively. *International journal of systematic and evolutionary microbiology* **64**, 926-932 (2014).

17. Roh, S. W. et al. *Sphingomonas aestuarii* sp. nov., isolated from tidal flat sediment. *International journal of systematic and evolutionary microbiology* **59**, 1359-1363 (2009).

18. Huang, H.-Y. et al. *Sphingomonas endophytica* sp. nov., isolated from Artemisia annua L. *International journal of systematic and evolutionary microbiology* **62**, 1576-1580 (2012).

19. Zhu, L. et al. *Sphingomonas gei* sp. nov., isolated from roots of Geum aleppicum. *International journal of systematic and evolutionary microbiology* **65**, 1160-1166 (2015).

20. Feng, G.-D. et al. *Sphingomonas gimensis* sp. nov., a novel Gram-negative bacterium isolated from abandoned lead–zinc ore mine. *Antonie van Leeuwenhoek* **105**, 1091-1097 (2014).

21. An, D.-S. et al. *Sphingomonas ginsengisoli* sp. nov. and Sphingomonas sediminicola sp. nov. International journal of systematic and evolutionary microbiology 63, 496-501 (2013).

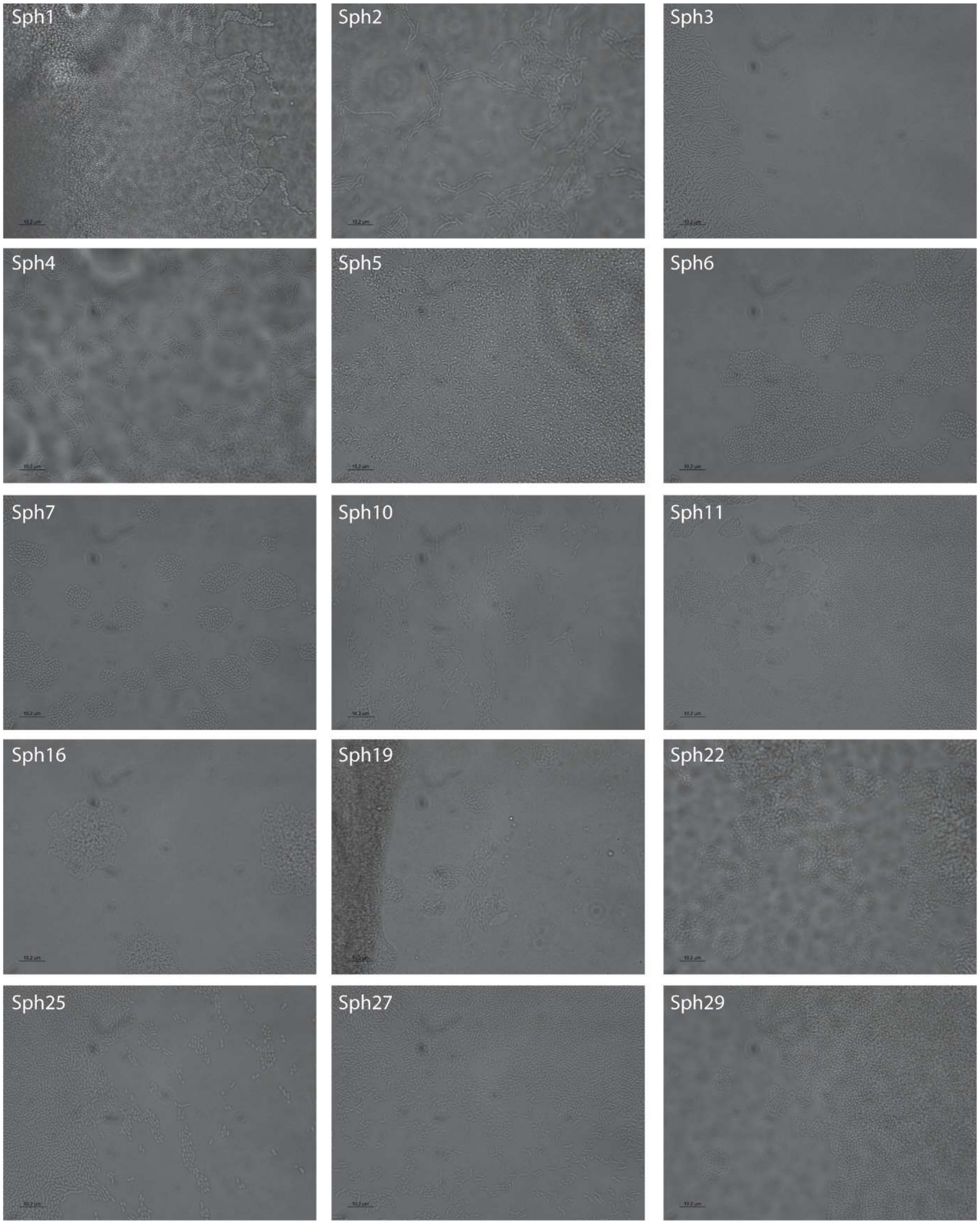
Sph1
Sph2
Sph3
Sph4
Sph5
Sph6
Sph7
Sph10
Sph11
Sph16
Sph19
Sph22
Sph25
Sph27
Sph29

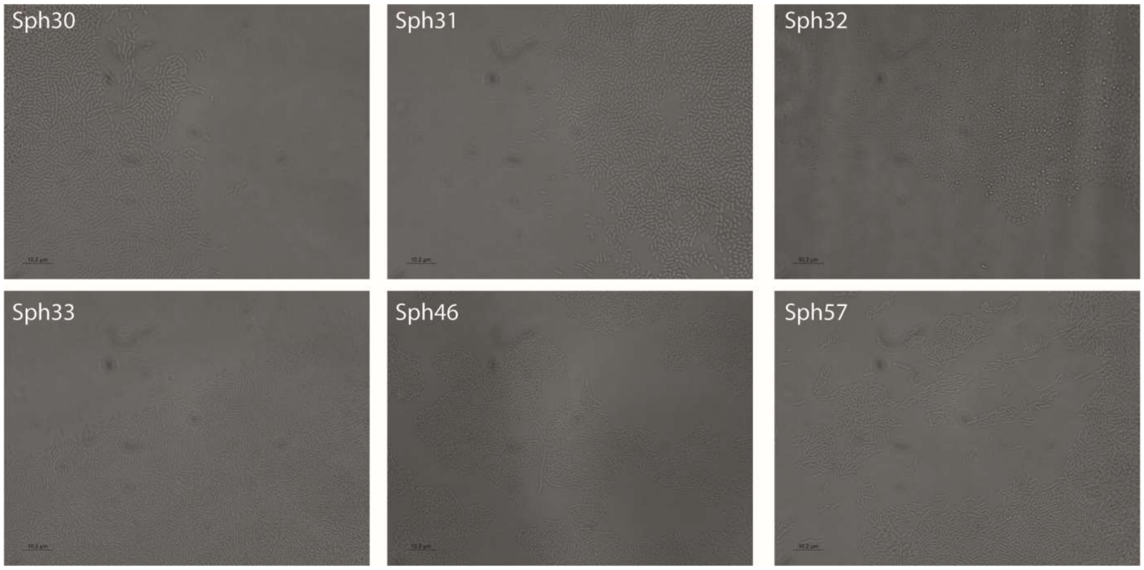

Figure S1: Phase contrast microscope micrographs of all Sph isolates twitching on the TMGG medium amidst of a microscopic slide after 24h of incubation

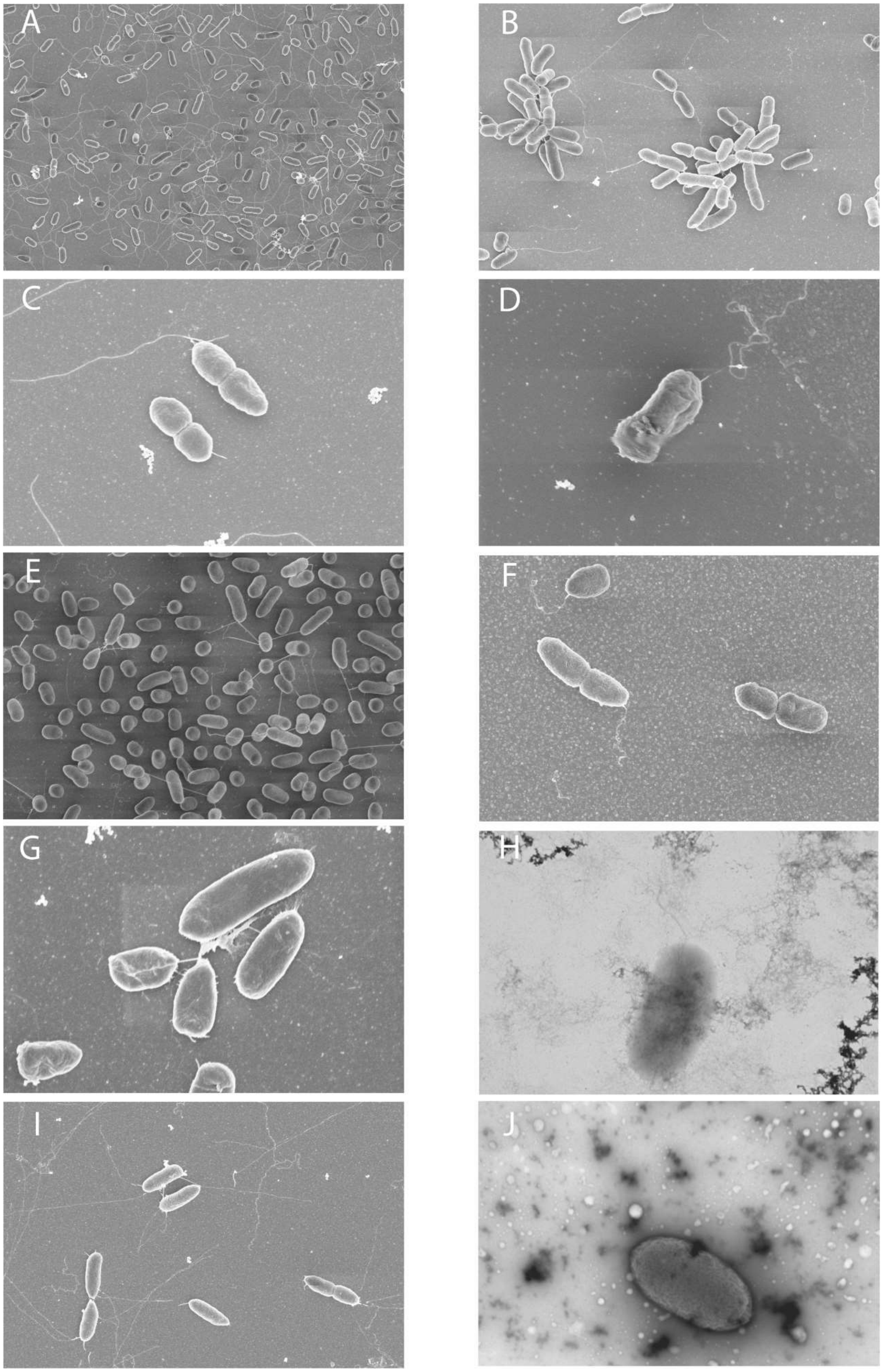

Figure S2. Electron microscope micrographs of the Sph isolate clades B, C, D, E, F, G, H, I, J and K.
